# Venomics Reveals Venom Complexity of the Piscivorous Cone Snail, *Conus tulipa*

**DOI:** 10.3390/md17010071

**Published:** 2019-01-21

**Authors:** Mriga Dutt, Sébastien Dutertre, Ai-Hua Jin, Vincent Lavergne, Paul Francis Alewood, Richard James Lewis

**Affiliations:** 1 Institute for Molecular Bioscience, The University of Queensland, St. Lucia, Queensland 4068, Australia; m.dutt@uq.edu.au (M.D.); a.jin@imb.uq.edu.au (A.-H.J.); p.alewood@imb.uq.edu.au (P.F.A.); 2 Institut des Biomolecules Max Mousseron, UMR 5247, Université Montpellier-CNRS, 34093 Montpellier, France; sebastien.dutertre@umontpellier.fr; 3 Léon Bérard Cancer Center, 28 rue Laennec, 69008 Lyon, France; vincent.lavergne@lyon.unicancer.fr

**Keywords:** conotoxin, *Conus tulipa*, intraspecific variation, venomics, transcriptomics, proteomics, conantokins, net hunting strategy, nirvana cabal, ion channel modulators

## Abstract

The piscivorous cone snail *Conus tulipa* has evolved a net-hunting strategy, akin to the deadly *Conus geographus*, and is considered the second most dangerous cone snail to humans. Here, we present the first venomics study of *C. tulipa* venom using integrated transcriptomic and proteomic approaches. Parallel transcriptomic analysis of two *C. tulipa* specimens revealed striking differences in conopeptide expression levels (2.5-fold) between individuals, identifying 522 and 328 conotoxin precursors from 18 known gene superfamilies. Despite broad overlap at the superfamily level, only 86 precursors (11%) were common to both specimens. Conantokins (NMDA antagonists) from the superfamily B1 dominated the transcriptome and proteome of *C. tulipa* venom, along with superfamilies B2, A, O1, O3, con-ikot-ikot and conopressins, plus novel putative conotoxins precursors T1.3, T6.2, T6.3, T6.4 and T8.1. Thus, *C. tulipa* venom comprised both paralytic (putative ion channel modulating α-, ω-, μ-, δ-) and non-paralytic (conantokins, con-ikot-ikots, conopressins) conotoxins. This venomic study confirms the potential for non-paralytic conotoxins to contribute to the net-hunting strategy of *C. tulipa.*

## 1. Introduction

Venomous animals have long been regarded as a valuable source of bioactive peptides that can have therapeutic potential, with several currently used clinically [1,2,3,4]. Marine cone snails produce relatively short cysteine-rich bioactive peptides called conotoxins that target various ion channels and receptors [5]. Amongst animal venoms, conotoxins arguably display the broadest suite of posttranslational modifications (PTM), which contribute to the broad spectrum of bioactivity in these exceptionally potent venoms [6]. To date, more than 800 species of snails in the genus *Conus* have been documented [7] with most species producing in excess of 1000 conotoxins [8]. As these molluscs are sluggish and small, it is clear that their complex venom arsenal has contributed to their success as predators [9,10]. To accelerate conotoxin discovery, there has been a shift from traditional assay-guided fractionation and Sanger sequencing to an increased use of next-generation sequencing (NGS) and proteomics as part of an integrated “venomics” approach [11]. Venomics has contributed to recent breakthrough in our understanding of the ecology and evolution of cone snails, including the role of defence in diet diversification [12] and biological messiness [13,14,15,16] in the accelerated diversification of conopeptides.

Cone snail venom composition appears to be affected by geography, diet and season [17], however, significant differences between individuals of the same species [8,18,19,20,21] make comparisons difficult and many earlier studies using pooled venom samples ignored the importance of venom variability [22]. Most well-studied *Conus* venoms have been isolated from fish hunting species that have evolved to target vertebrates [5,23]. The piscivorous cone snail *Conus tulipa* is classified phylogenetically in the *Gastridium* clade along with the closely related and potentially lethal *Conus geographus* [24]. Whereas this study follows the phylogeny published by Puillandre et al. [7], where the *Gastridium* species represents a subgenus within the single genus *Conus*, other authors have suggested that *Gastridium* should be recognized as a separate genus [25]. *C. tulipa* (50–80 mm) is smaller than *C. geographus* (80–120mm) but both possess a thin fragile shell and are generally considered the deadliest cone snails to humans [24]. Nevertheless, no systematic characterization of *C. tulipa* venom has been reported, with only ρ-TIA [26,27,28], µ-TIIIA [29], conantokin-T [30], conotoxin TVIIA [31] and conopressin-T [32] characterized to date. *C. tulipa* utilises a net-feeding strategy, exclusive to this clade, which involves enlargement of the snail’s rostrum and secretion of conotoxin(s) presumably through the proboscis to suppress the escape response of fish facilitating prey capture by mouth. Olivera et al. coined the ‘nirvana cabal’ [22] to describe the chemistry that facilitates net feeding, although the bioactive components of the nirvana cabal remain to be fully characterized.

Using 454 pyrosequencing combined with top-down and bottom-up mass spectrometry and dedicated bioinformatic tools, we characterized the complex mixture of venom peptides that comprise the venom of *C. tulipa*. Insights into intraspecific venom peptides were obtained by comparing the transcriptomes of two specimens of *C. tulipa* collected from the same geographical location. This systematic characterization broadens our understanding of the venom peptides contributing to the predatory and defensive behaviour of *C. tulipa.*

## 2. Results

### 2.1. Transcriptomic Intraspecific Variation

**Figure 1:**
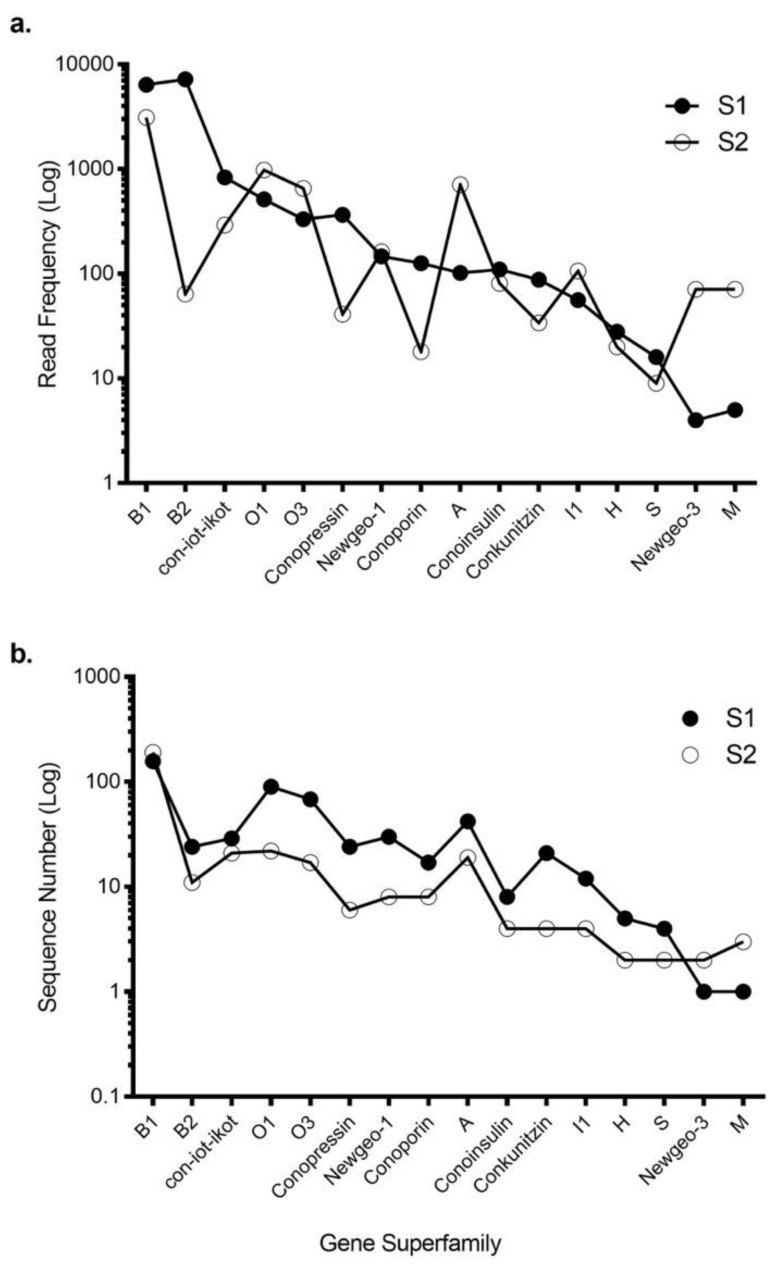
Transcriptomic expression of gene superfamilies for S1 and S2. **(a)** The conopeptide precursor read frequency and **(b)** the number of unique conotoxin sequences expressed by each specimen across the 16 common gene superfamilies. These data highlight the variable read frequency, with S1 expressing significantly more unique sequences across all superfamilies except the newgeo-3 and M superfamilies.

Using 454 sequencing, ConoSorter and manual editing, we generated venom gland transcriptome databases for two *C. tulipa* specimens, S1 and S2. The S1 intact venom duct yielded 522 conotoxin precursors that clustered into 16 known gene superfamilies (Table S1), while stripped venom duct of S2 yielded fewer (328) conotoxin precursors that clustered into 18 known gene superfamilies (Table 1 and Table S1). Overall, 16 gene superfamilies were common to both specimens (Table 1, Figure 1), with superfamily B1 encoding conantokins dominating the venom gland transcriptome of both specimens. In addition, gene superfamilies B2, O1, O3, con-ikot-ikot, conopressins and conoporins had high expression levels in both specimens (Figure 1). No novel gene superfamily was identified.

**Figure 2:**
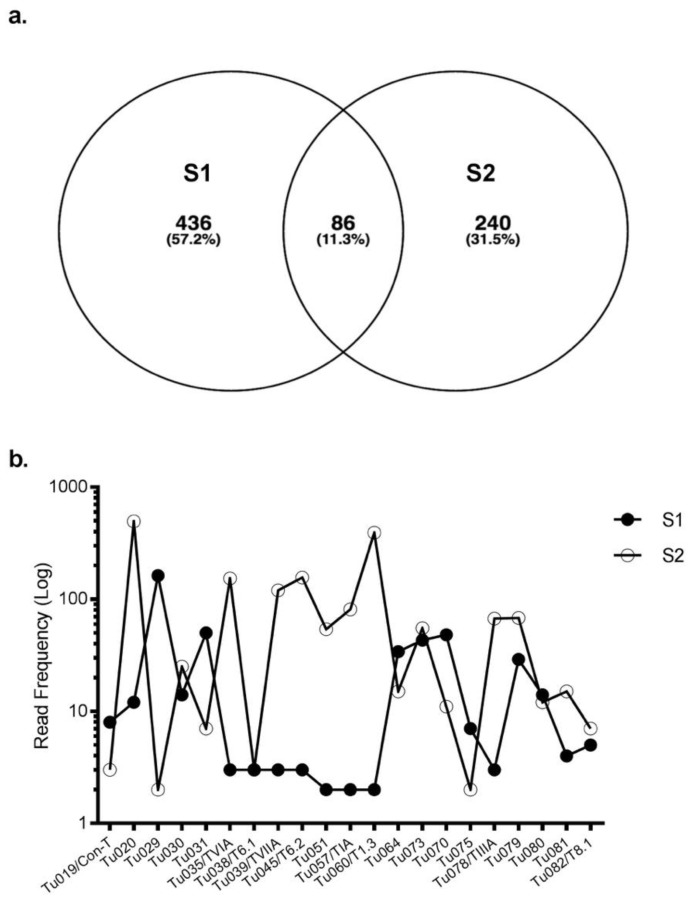
Expression of common precursors in S1 and S2. **(a)** A Venn diagram representing the total number of precursors found in S1 and S2. Both specimens commonly expressed 86 conopeptide precursors. **(b)** Expression level of the common precursors in S1 and S2. For superfamilies that shared >10 precursors (eg, B1, O1, O3), data is shown for the highest expressing precursor for each specimen in that superfamily. For known venom peptides, their name follows the transcript nomenclature.

Eighty-six conotoxin precursors (Tu001–Tu086) were common to both specimens, comprising ~ 10% of the total identified conotoxin precursors. Of these, 29 belonged to B1, 2 to B2, 11 to O1, 6 to O3, 8 to A, 8 to con-ikot-ikot, 4 to conopressin-conophysin and 6 to the recently described newgeo-1 superfamily from *C. geographus* (Table S2). Interestingly, most precursors had widely varying read frequencies between the specimens. For example, precursor Tu029 was the highest expressing superfamily B1 precursor in S1 (162 reads) but one of the lowest expressing (2 reads) in S2. In contrast, precursor Tu020 was highly expressed in S2 (495 reads) but had only 12 reads in S1 (Figure 2). Overall, 41 of 86 common conotoxin precursors had read levels that were within 3-fold difference (Table S2).

### 2.2. Proteome Analysis

**Figure 3:**
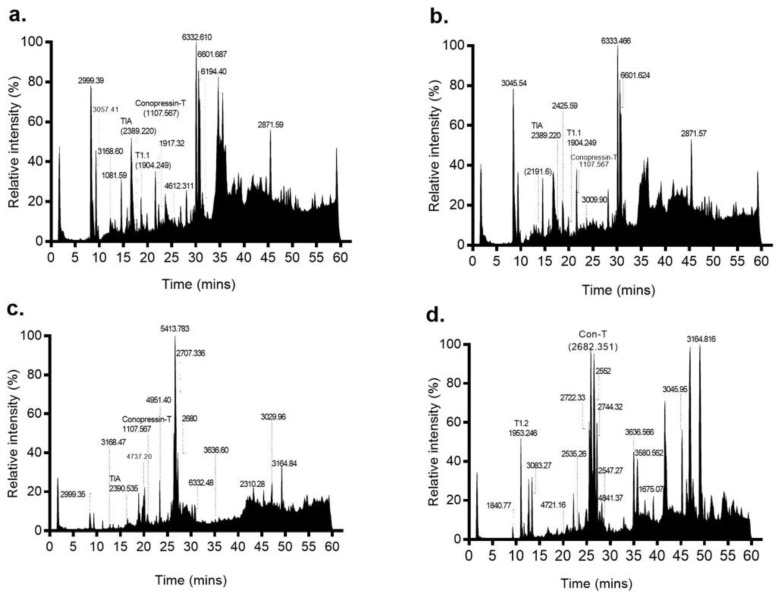
Mass profiles of the four venom duct segments of *C. tulipa*. LC-MS profiles of (**a**) proximal, (**b**) proximal central, (**c**) distal central and (**d**) distal sections of the venom duct are shown, with prominent masses indicated. The mass profiles for the proximal and proximal central sections were similar and included three known conotoxins ρ-TIA (relative intensity 10.2%), T1.1 (relative intensity 0.17%) and conopressin-T (relative intensity 57.5%). In contrast, the mass profiles for the distal and distal central sections are quite different, with the latter displaying a much simpler peptide profile dominated by the peptide mass 5413.783 Da, while the distal section was dominated by Con-T (2682.351 Da) along with peptide masses 2722.33 Da, 2744.32 Da and T1.2. Additionally, relatively low expression of conopressin-T (4.45%) and ρ-TIA (0.1%) peptides was observed within the DC section.

**Figure 4:**
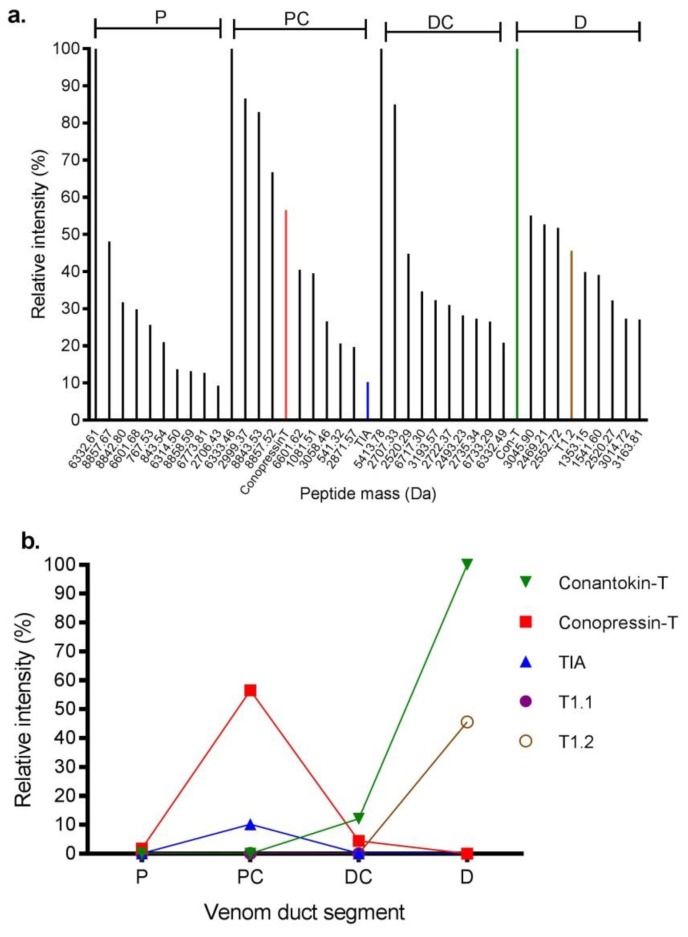
Relative intensity of prominent venom peptides expressed in different sections of the *C. tulipa* venom duct. **(a)** Relative intensity of the ten dominant peptides from the proximal (P), proximal central (PC), distal central (DC), and distal (D) venom duct sections. Conopressin-T (red bar), ρ-TIA (blue bar) and conantokin-T (green bar) were amongst the major expressing peptides analysed in the proteome **(b)** Relative intensity of known *C. tulipa* peptide masses across the venom duct sections. Conantokin-T (2682. 3514 Da) dominated the D section, while conopressin-T (1107. 5675 Da) and ρ-TIA (2389.2206 Da) had relatively high expression levels in the PC sections. T1.2 (1953.2469 Da) also had relatively high expression in the D section, while T1.1 (1904.2497 Da) showed relatively low expression across the venom duct.

LC-MS analysis was performed on the proximal (P), proximal central (PC), distal central (DC) and distal (D) sections of the S2 venom duct (Figure 3). The P and PC sections had similar profiles, with twelve common masses shared between the two sections. In contrast, the DC and D sections were each quite distinct, with the DC section producing only a few easily detectable masses, while the D section was more complex. Through a combined top-down and bottom-up proteomics approaches, we confirmed the presence of 7 of 9 reported *C. tulipa* venom peptides (Table S3). These include ρ-TIA (2389.22 Da), conopressin-T (1107.567 Da) and T1.1 (1904.249 Da) in the P and PC sections (Figure 3), though low levels of the former were also identified in the DC section (Figure 4). In the D section, the dominant mass was conantokin-T (2682.351 Da) and the moderately expressing T1.2 (1953.246 Da) (Figure 4). Tandem MS/MS analysis of the duct sections detected and confirmed the presence of 18 conotoxins (Table 2).

### 2.3. Transcriptomic Variance Within Gene Superfamilies

#### 2.3.1. Superfamily B1 [33]

Superfamily B1 dominated expression in both the specimens, with a total of 377 precursors recovered, including 157 unique precursors in S1 and 191 unique precursors in S2, together with 29 common precursors (Tu001–Tu029). Conantokin-T has been well-characterized [33] but its gene precursor sequence has remained incomplete. From our transcriptome analysis, we established the full precursor sequence of conantokin-T (Tu020), with most other B1 superfamily transcripts being variants of conantokin-T (Table S1). MS/MS analysis matched peptide fragments belonging to Tu020 (495 reads) from S2 found in the distal venom duct, confirming venom expression of conantokin-T (2682.35 Da) (Table 2).

#### 2.3.2. Superfamily B2 [14]

In *C. tulipa*, twenty-four B2 precursors were identified in the transcriptome of S1 at moderate transcription levels. Of the thirteen precursors identified in S2, MS/MS analysis only detected fragments belonging to a single conotoxin precursor (Tu030, 25 reads) found in the PC and P sections of the venom duct (Table 2). B2 peptides are cysteine poor, similar to the superfamily B1 conantokins (Table S4). Only two B2 conotoxin precursors (Tu030 and Tu031) were common to both specimens (Table S2).

#### 2.3.3. Superfamily O1 [34]

S1 and S2 expressed 83 and 11 unique superfamily O1 precursors, respectively, with eleven precursors (Tu032–Tu042) common to both specimens (Table S2). The O1 precursors expressed in S1 and S2 could be classified into four major subtypes based on their propeptide sequences, including precursors for TVIA, TVIIA, putative T6.1 and a putative new peptide sequence named T6.2 (Table S4). Despite 23 precursors encoding for TVIA in S1 and 7 in S2, only two precursors (Tu034 and Tu035) were common to both (Table S2). For TVIIA, we found two precursors common to both S1 and S2. The MS/MS analysis detected fragments matching to two precursors encoding for TVIA (Tu035, 153 reads and Tu274, 376 reads) and a fragment matching precursor encoding for a putative T6.1 toxin (Tu032, 3 reads) in the PC section (Table 2).

#### 2.3.4. Superfamily O3 [35]

Fifty-eight unique superfamily O3 precursors from S1 and 12 from S2 were identified, while six precursors (Tu043–Tu048) were common to both specimens. All precursors had the same signal sequence MSGLGIMVLTLLLLVLMTTSH, and depending on their mature peptide sequence differences, superfamily O3 precursors were classified into two groups; T6.3 (SAKGTVSWRKKHCCCIRSNGPKCSRICIFKFWC) that displayed 92% similarity to G20 [35], and T6.4 (CEMQCEQKKKHCCRVREERIQCAPKCWGIEW) that was 90% similar to G6.6 (Table S4) [32]. MS/MS fragments matching two T6.4 precursors (Tu297, 51 reads and Tu298, 76 reads) were detected in the PC venom duct sections of S2 (Table 2).

#### 2.3.5. Superfamily A [36]

S1 expressed thirty-three unique superfamily A precursors and S2 eleven unique precursors, with eight precursors (Tu049-Tu056) common to both (Table S2). Superfamily A conopeptides included ρ-conotoxin TIA and putative α-conotoxins T1.1 and T1.2 (Table S4). Broadly, the analysed precursors could be classified into four subgroups depending on their mature sequence, (Table S4). The new peptide T1.3 (Tu056, 391 reads) displayed high similarity to peptide G1.9 from *C. geographus* [37], with MS/MS detecting a fragment matching T1.3 in the DC venom duct section (Table 2). 15 ρ-TIA precursors from S1 and two from S2 were detected, with S1 additionally expressing 11 C-terminal sequence variants of ρ-TIA (Table S5). A single ρ-TIA transcript (Tu053) was common to both the specimens, with 2 reads in S1 and 81 reads in S2 (Table S2). A mass corresponding to ρ-TIA (2389.22 Da) was detected by LC/MS at modest levels in the P, PC and DC venom duct sections of S2 (Figure 3; Figure 4), although no MS/MS fragments of ρ-TIA could be detected. In addition to ρ-TIA, a mass corresponding to T1.1 (1904.249 Da) was detected by LC/MS in the P and PC sections, while T1.2 (1953.246 Da) was detected with relatively high intensity in the distal venom duct section that was confirmed by an MS/MS match to Tu051 (Table 2) providing the first proteomic evidence for T1.1 and T1.2 (Table S4).

#### 2.3.6. Conopressins-Conophysin [22]

Conopressin-conophysin precursors were analysed from the venom duct transcriptomes of both specimens. Mature conopressin-T was associated with 20 unique conopressin precursors in S1 and 6 unique precursors in S2, with four common (Tu057–Tu060) to both (Table S2). Of these, three precursors (Tu057–Tu059) displayed a mature conophysin sequence that was 93% similar to conophysin-G [38]. From the proteome, we identified a mass corresponding to conopressin-T (1107.567 Da) expressed in the P, PC and DC sections of the venom duct of S2 (Figure 3), with a relatively high expression (57%) in the PC sections (Figure 4). Venom expression of conopressin-T was confirmed by MS/MS.

#### 2.3.7. Con-ikot-ikots [37]

S1 expressed 20 and S2 expressed 11 con-ikot-ikot precursors, with eight precursors (Tu061–Tu068) common to both specimens (Table S2). This class of conotoxins is associated with different signal peptide regions and remain to be assigned to a specific superfamily. In this study, most of the expressed precursors displayed the signal sequence MAMNMSMTLSTFVMVVVAAT, similar to the signal sequence of con-ikot-ikots from *C. striatus*, despite their mature peptide region being similar to con-ikot-ikots isolated from *C. geographus* [39] (Table S3). MS/MS analysis confirmed the presence of two con-ikot-ikot peptide fragments (Tu065, 11 reads;Tu068, 28 reads) in the D and DC venom duct sections of S2 (Table 2).

#### 2.3.8. Conoporins [40]

Three conoporin precursors (Tu069–Tu071) were common between both specimens, while S1 expressed fourteen and S2 expressed five unique precursors. MS/MS detected fragments that matched to two precursors (Tu314 with 3 reads and Tu0316 with 2 reads) across the venom duct sections of S2 (Table 2).

#### 2.3.9. Superfamily M [41]

μ-TIIIA from *C. tulipa* is a potent inhibitor of mammalian neuronal sodium channels [41]. Surprisingly, superfamily M expression levels were low in the S1 and S2 transcriptomes (1 and 3 precursors, respectively), with all precursors being identical to TIIIA (Table S1) although the μ-TIIIA mass was not detected in the proteome. One precursor (Tu072) was common to both specimens (Table S2).

#### 2.3.10. Conoinsulin [42]

S1 had 8 conoinsulin precursors and S2 expressed 4 precursors, with only a single precursor (Tu073) common to both (Table S2). All precursors had the signal sequence MTTLFYFLLMALGLLLYVCQSSFGNQ and comprised both the A and B chains (Table S1). No mass corresponding to conoinsulins could be identified from the proteomic analysis.

#### 2.3.11. Conkunitzins [43]

Nineteen and 3 unique conkunitzin precursors were analysed in S1 and S2, respectively, with two precursors common to both (Tu074 and Tu075). Despite low transcriptomic expression, MS/MS analysis detected fragments that matched to two conkunitzin precursors (Tu320, 2 reads and Tu075, 12 reads) in the DC and PC venom duct sections respectively (Table 2).

#### 2.3.12. Superfamily S [44]

A single common superfamily S precursor (Tu077) that was 91% similar to G8.3 from *C. geographus* [44] was identified in S1 and S2 (5 and 7 reads, respectively). The novel precursor had the signal sequence MMSKMGAMFVLLLLFTLASSQ and a disulfide connectivity belonging to framework VIII (10 cysteine residues, -C-C-C-C-C-C-C-C-C-C-). We have named it as putative T8.1 (Table S4) since no mass corresponding to superfamily S precursors could be detected in the proteome.

#### 2.3.13. NewGeo-1 [12]

For both specimens, five precursors were identified (Tu081–Tu086) that displayed the signal sequence MSRLFLILLVIAVITLKADAS and were devoid of cysteine residues in the mature region. S1 also had 24 additional precursors and S2 had two additional related precursors. No masses associated with this superfamily were detected in the proteome.

### 2.4. Intra-Clade Transcriptomic Comparison

**Figure 5:**
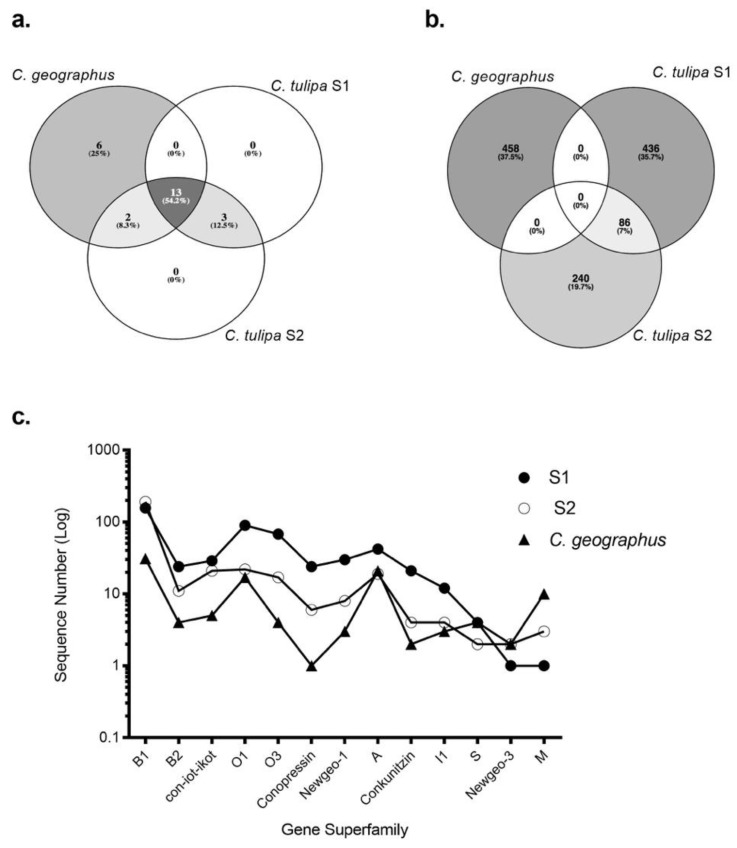
Intra-clade transcriptomic comparison for the *Gastridium* clade. Three-way Venn diagrams depicting the extent of the **(a)** gene superfamily and **(b)** venom duct conotoxin precursor overlap observed between *C. geographus* and *C. tulipa* (S1 and S2). Thirteen gene superfamilies were common to both the species, while six and three superfamilies were exclusive to *C. geographus* and *C. tulipa* respectively. Despite this broad overlap, both species produce a cohort of conotoxin precursors that are distinctly exclusive. **(c)** The number of conotoxin sequences expressed by both species across the 13 common gene superfamilies.

To analyse the extent of clade-specific conotoxin expression, the transcriptomes of S1 and S2 were compared to their closest relative, *C. geographus* [12]. The transcriptomes of both species have been generated using 454 pyrosequencing platform. Remarkably, thirteen gene superfamilies, including B1, B2, A, O1, con-ikot-ikot and conopressin-conophysin, were jointly expressed within the *Gastridium* clade (Figure 5). Despite this broad overlap, no common conotoxin precursor was analysed between *C. geographus* and *C. tulipa*, with each species displaying a unique venom duct transcriptome (Figure 5b). S2 shared two superfamilies, O2 and T, with *C. geographus*, while no superfamily was exclusively common to S1 and *C. geographus*. Additionally, *C. geographus* expressed six superfamilies exclusively, including I3, J, contryphans and contulakins (Table S6). Three superfamilies, H, conoinsulins and conoporins, were found exclusively to *C. tulipa.* While no transcriptomic evidence for conoinsulins and conoporins was reported in the transcriptomic study for *C. geographus* [12], these conotoxins have previously been detected in the venom gland of *C. geographus* [42,45]. The numbers of conotoxin precursors, across the common superfamilies, were analysed to be 10-fold higher in the *C. tulipa* venom duct transcriptome.

## 3. Discussion

Venomic studies are increasingly being used to develop comprehensive understandings of animal venoms. In this study, we describe the first venomic analysis of the piscivorous net hunter *C. tulipa* using a combination of 454 pyrosequencing, advanced mass spectrometry and dedicated bioinformatic tools. In addition, we evaluated the intra-specific mRNA variation across two *C. tulipa* specimens to better understand venom variation in Conidae. Transcriptomics uncovered 764 conotoxin precursors that were classified into 16 known superfamilies across the two specimens, with two additional superfamilies identified as unique to one specimen. Despite this superfamily overlap, 10% of the identified conotoxin precursors were found in both specimens, representing the venom ‘fingerprint’ for *C. tulipa*, and establishing that much of the dramatic proteomic variation previously reported [12] arises at the mRNA level [8,19,21,46,47,48]. Overall, the venom of *C. tulipa* is characterized by the expression of non-paralytic peptides previously hypothesized to contribute to the nirvana cabal of net hunting *Conus* species [22]. Amongst these, superfamily B1 (conantokins) was most abundant in the distal venom duct proteome, whereas conopressin-T was most abundant in the proximal venom duct, suggesting non-paralytic conotoxins may play distinct and separate roles in prey capture and/or defence in *C. tulipa*.

While geography and diet are believed to contribute to the observed venom variation between cone snail species and individuals [13,14,16], our transcriptomic comparison revealed quite distinct venom peptide repertoires despite both the specimens being collected from the same geographical region. Despite this remarkable variability, the top five gene superfamilies expressed in both *C. tulipa* specimens were B1, B2, O, A and con-ikot-ikot. Specimen S1 expressed ~ 2.5 times more conotoxin precursors than S2, with most being variants of the parent precursor. These variants were observed to be arising due to insertion/deletion of precursor sequences, an elongated post-cleavage sequence, and changes in 1-2 amino acids (in either propeptide or mature peptide); synonymous to the mRNA messiness that has been previously described [13]. For example, while the venom duct transcriptome of S2 expressed only a single variant of ρ-TIA, in S1 we analysed eleven variants of the ρ-TIA peptide (Table S5). Another contributing factor to this observed peptide variation is variable peptide processing previously identified as expanding observed conopeptide diversity [14]. These peptide variants create a diverse sequence library allowing enhanced forms to be selected in response to changing evolutionary pressures. Differences in sample preparation could also contribute to the observed intraspecific difference, with the whole venom duct retaining the epithelial cell lining [49,50] that might contribute to the increased number of peptide precursors identified in S1. Injected venom has also shown prominent intraspecific differences [19], however, we were unable to obtain injected venom from our live specimens to investigate milked venom variation in *C. tulipa*.

This study revealed that non-paralytic conotoxins dominated the transcriptome of *C. tulipa*, including conantokins, conopressins, conkunitzins, con-ikot-ikot and conoinsulins previously proposed to contribute to the nirvana cabal [51]. Conantokins dominated the distal venom duct, suggesting a role in predation [12] and potentially reflecting deployment during net hunting by *C. tulipa*. However, despite a suggested predatory role, conopressin-T dominated the proximal and proximal central venom duct sections previously shown to contribute to the defensive venom in the closely related *C. geographus* [45]. Therefore, conopressin-T may play a role in defense [12], perhaps reflecting its shift to an antagonist of the vasopressin receptor [32]. Conoinsulins have been recently implicated in the nirvana cabal of *C. geographus* [42], however, their transcriptomic expression was low and could not be detected in the proteome, suggesting these modified hormones likely play no more than a limited role in *C. tulipa* venom. ρ-TIA and the conkunitzins were also detected at moderate levels in distal venom duct sections and thus potentially contribute to the predatory strategy of *C. tulipa.* Whereas ρ-TIA injected into fish was non-lethal [27], its contribution to the nirvana cabal remains to be evaluated. Conkunitzins have been characterized to block human K_V_1.7 [43], associated with the functioning of the β-cells of Langerhans and thus may cause hypoglycaemia and potentially synergise with conoinsulins as part of the nirvana cabal [52] but effects on fish and any synergy remains to be determined. In contrast, α-conotoxins (T1.1) and ω-conotoxins (TVIA, T6.1, T6.4) were identified in the proximal venom duct, suggesting these conotoxins contribute to a defensive strategy, while α-conotoxin T1.2 was detected in the distal venom duct, suggesting an involvement in *C. tulipa* predatory venom [12]. In addition, superfamilies O3, H and A were typically found in the distal central section. Given net feeders can also deploy a hook and line strategy to catch fish [12], it is plausible that their venom ducts have specialized sections for both predatory modes in addition to a specialized section for defence.

Comparing the transcriptomes of *C. tulipa* and *C. geographus* revealed these species had thirteen common gene superfamilies (Figure 5 and Table S6) [12], including high levels of the non-paralytic conantokins in the distal venom duct proteome. These findings support the view that cone snails belonging to the same evolutionary clade display similar venom profiles and functions that have arisen due to their overlapping predatory strategies [53]. However, both species have developed a unique venom peptide arsenal indicative of divergent evolution. This study showed that *C. tulipa* produced approximately 10-fold more conotoxin precursors than *C. geographus* reflecting the peptide expression at the time of mRNA preparation and are likely to vary across *C. tulipa* specimens. Both *C. tulipa* and especially *C. geographus* are considered dangerous to humans, with *C. geographus* implicated in numerous fatal stings [18]. *C. geographus* has a complex proximal venom duct profile dominated by ion channel modulating paralytic venom peptides [12,18]. Like *C. geographus,* we identified transcripts for superfamily A, M, O1 and O3 precursors that translate to putative paralytic α-, μ- and ω-conotoxins targeting the nicotinic acetylcholine receptors, voltage-gated sodium and calcium channels, respectively.

## 4. Materials and Methods

### 4.1. Transcriptome Analysis

#### 4.1.1. Venom Collection, mRNA Extraction, cDNA Library, 454 Pyrosequencing and Assembly

Due to their low abundance at the collection site, we were limited to a small sample size for this study. Two adult *C. tulipa* specimens (measuring 60 mm) were collected from Lady Musgrave Island, Queensland, Australia under GBRMPA permit number G13/36201.1 and dissected on ice. The intact venom duct of Specimen 1 (S1) and stripped venom duct of Specimen 2 (S2) were treated with 1 mL TRIzol^®^ reagent (Thermo Fisher, Scoresby, VIC, Australia), and the total RNA extracted following the manufacturer’s instructions (Invitrogen, Carlsbad, CA, USA). Total RNA was further treated with an Oligotex Direct mRNA mini kit (Qiagen, Valencia, CA, USA) to extract mRNA. The extracted mRNA was sent for 454 pyrosequencing using a Roche GS FLX Titanium (Roche Diagnostics, Indianapolis, IN, USA) sequencer platform (one/eighth of a plate per sample) at the Australian Genomic Research Centre (University of Queensland, Brisbane, Australia). The corresponding contig assembly was obtained using Newbler 2.3 (Life Science, Frederick, CO, USA).

#### 4.1.2. Conopeptide Sequence Analysis

Raw cDNA reads and the assembled contigs obtained from S1 and S2 were searched, filtered and clustered into different gene superfamilies by our in-house programme ConoSorter [54]. The precursor list was trimmed to remove sequences < 50 amino acids (most conotoxin precursors are 70-100 a.a. long), single reads, sequence containing ambiguous amino acids (“X”), redundant sequences, and those with signal peptide sequences displaying < 40% hydrophobicity. Unsorted precursors were checked with SignalP 4.0 [55] and precursors with a valid signal sequence clustered into different gene superfamilies. Propeptide regions, mature peptide cleavage sites, cysteine frameworks and likely posttranslational modifications (PTMs) were identified using the ConoPrec tool in ConoServer database [56]. Precursors that could not be classified using ConoPrec were considered as candidate novel gene superfamilies. Finally, all selected conopeptide precursors were submitted to a BLASTP search with default parameters and assessed for their similarity to known conopeptide precursors in the UniProt/BLAST database [57] and all housekeeping-related genes were removed. The minimum number of reads constituting a valid precursor sequence was set at 2.

#### 4.1.3. Conotoxin Nomenclature

Two separate transcriptomes have been described in this study and thus we have serially numbered the common precursors first, and then continued the numbering for the unique precursors identified in each specimen. The identified common conotoxin precursors have been numbered as Tu001–Tu086. The identified unique precursors from each specimen have been numbered as Tu087–Tu522 for S1, and Tu087–Tu328 for S2. Nomenclature of putative new conotoxins precursors has followed the conventional nomenclature [58], wherein the identifying species is indicated with a capital letter, followed by numerical representation of the disulfide framework.

### 4.2. Proteome Analysis

#### 4.2.1. Liquid Chromatography-Mass Spectrometry (LC-MS)

Prior to mRNA extraction, the crude venom from the venom duct sections of S2 was collected for proteomic analyses, as previously described [14]. Briefly, S2 was placed on ice before the venom duct was removed by dissection. Venom duct from S2 was dissected into four segments: proximal (P), proximal central (PC), distal central (DC) and distal (D) relative to the venom bulb. The dissected venom was collected by gently squeezing and stripping the duct segments with forceps. The collected venom samples were lyophilized and dry weight determined. 1 µg/µL of each soluble crude venom extract was subjected to top-down LC-MS on a SCIEX 5600 Triple TOF MS/MS mass spectrometer (Framingham, MA, USA) and a list of detected masses was generated, as previously described [14].

#### 4.2.2. Enzyme Digestion

Fifty µL (1 µg/µL) protein samples were subjected to reduction and alkylation using 2% *v*/*v* iodoethanol and 0.5% *v*/*v* triethylphosphine respectively, as per Hale et al. [59]. The denatured proteins were subjected to enzymatic digestion with sequencing grade porcine trypsin (Promega, Auburn, VIC, Australia). Trypsin was activated in 40 mM NH_4_HCO_3_ buffer, pH 8.0 and a ratio of 1:100 (w/w) of enzyme to protein was used. Samples were incubated overnight at 37 ^°^C and each digest was completed by heating the sample for 4 min on the lowest microwave power setting.

#### 4.2.3. Liquid Chromatography-Electrospray Ionization Mass Spectrometry/Mass Spectrometry (LC-ESI-MS/MS)

The reduced, alkylated and enzyme-digested peptides were subjected to bottom-up tandem mass spectrometry on the SCIEX 5600 Triple TOF mass spectrometer, coupled to a Shimadzu 30 series HPLC. Information Dependent Acquisition (IDA) was performed on the samples as previously described [14]. The detected peptide fragments, including those with known PTMs were matched to the precursor sequences on the generated *C. tulipa* transcriptome database using the ProteinPilot v4.0.0 software (SCIEX, Framingham, MA, USA). The mass tolerance for precursor ions was set at 0.05 Da and 0.1 Da for the fragment ions. Peptide fragments with > 99% confidence intervals were selected as matching to the transcriptomic sequence.

### 4.3. Data Visualisation

The presented multi-omics data was visualised using Prism software, v7.0.0 (GraphPad, La Jolla, CA, USA). Venn diagrams were generated using Venny v2.1.0 software (Spanish National Centre for Biotechnology, Madrid, Spain). [60]

## 5. Conclusions

Through a combination of next generation sequencing and advanced proteomics, we have identified 764 conotoxin precursors across two specimens of *C. tulipa*. Amongst these, the dominant expression of non-paralytic peptide classes, including conantokins, conopressins and con-ikot-ikots, reaffirm their putative involvement in the net hunting predation strategy. Additionally, the possible involvement of ρ-TIA and conkunitzins in the predation-evoked venom requires further investigation in suitable prey systems to help establish their role in prey capture. Finally, through this study we have expanded our understanding of the venom profile of the *Gastridium* clade that includes two of the most lethal cone snails known to man.

## Figures and Tables

**Table 1:** Transcriptomic variation between two *C. tulipa* specimens.

Transcriptome Features	Specimen 1 (S1)	Specimen 2 (S2)
454 raw reads generated	100,564	33,516
Number of final conotoxin precursors	522	328
Number of gene superfamilies	16	18
Total read frequency (level of transcription)	16,333	6426

**Table 2:** Novel conotoxin precursors identified in the four venom duct sections of *C. tulipa* by LC-MS/MS at 99% confidence.

^1^ Section	Matched Precursor (S2)	MS/MS Fragment	PTM	Precursor (Da)	z
D	Tu0051/T1.2	SNPACAGNNPH	Ala->Gly@5	1169.430	2
D	Tu316	AIASSVVTPGSSMK		1333.691	2
D	Tu068	MINAETQTR		1062.510	2
D	Tu065	NCMLINVQQLGLR	Asn -> Thr @1, carbamidometh@2	1544.830	2
D	Tu020	MLENLREAEVK	Carboxy(E)@3	1374.681	3
DC	Tu076	ADRDTDPDDENPR	Oxidation(P)@7	1530.618	3
DC	Tu056/T1.3	VKDFK		635.364	2
DC	Tu316	AIASSVVTPGSSMK		1333.691	2
DC	Tu065	NCMLINVQQLGLR		1524.820	2
DC	Tu068	MINAETQTR		1062.500	2
DC	Tu314	DLADTRYR	Arg -> Ser @ 6	939.4289	2
DC	Tu320	AAFHMFYFDQFSK		1637.730	3
PC	Tu298/T6.4	DGTGQCAPK	Gln->Asp@5, Carbamydomethyl @ 8, oxid(P)@10,	1190.560	3
PC	Tu297/T6.4	VRDNR		658.351	2
PC	Tu032/T6.1	DALKNLK		800.457	2
PC	Tu035/TVIA	SCNPYSR	Carbamydometh@2, Deamidated(N)@3, Oxidation(P)@4,	899.342	2
PC	Tu274/TVIA	ALKNLKDSRGGSAR	Deamidated(R)@8.	1474.787	2
PC	Tu314	VVTSGSSLQGTSLK		1362.730	2
PC	Tu075	VFIYGGCDGNANR		1428.640	2
PC	Tu316	AIASSVVTPGSSMK		1333.690	2
PC	Tu059/conopressin-T	NLDNIEGH		910.413	2
PC	Tu296/T6.4	VRDNR		658.340	2
PC	Tu314	GIASKVVTSGSSLQ		1332.830	2
PC	Tu030	VPEDASNLQGFDQG		1475.780	2
P	Tu030	LPFNNVEGATNDLG QFEPSAENEDGKFRFF		1475.650	2
P	Tu076	LTLSAPK	Deam @ 3	728.443	2
P	Tu075	AAFHMFYFDQFSK		1637.735	3
P	Tu059/conopressin-T	NLDNIEGH		910.414	2
P	Tu314	FALKNPVLQINSGVTTSTPTGIEPGK	Deamidated(N)@5; Deamidated(N)@11; Ser->Gly@12	2640.405	3
P	Tu314	AVRAIASSVVTPGSSMKGGPLK		2112.360	3
P	Tu314	VVTSGSSLQGTSLKDLADTRYRVTCAIQVENWTK		2321.149	3

^1^ Distal (D), Distal Central (DC), Proximal Central (PC) and Proximal (P).

## Supplementary Materials

The following are available online at http://www.mdpi.com/1660-3397/17/1/71/s1, Table S1: Unique transcriptomic precursors from S1 and S2, Table S2: Common transcriptomic precursor sequences, Table S3: *C. tulipa* peptides reported in literature and their evidence from the proteome investigation, Table S4: List of the major gene superfamilies expressed in the venom duct of S1 and S2, Table S5: List of the ρ-TIA precursor variants analysed from the S1 transcriptome, Table S6: Venom duct transcriptome expression of gene superfamilies in *C. tulipa* and *C. geographus*.
