# Supplementary material for: Venomics Reveals Venom Complexity of the Piscivorous Cone Snail, Conus tulipa
Source: Mar Drugs. 2019 Jan 21;17(1):71. doi: 10.3390/md17010071 (PMC6356538; doi:10.3390/md17010071)
Supplement: Supplementary file 1 [file marinedrugs-17-00071-s001.zip › marinedrugs-410587 suply for XML conversion/marinedrugs-410587 supply Tables.pdf]

Supplementary Tables

S3 Table. ***C. tulipa*** **peptides reported in literature and their evidence from the proteome investigation**. Information has been provided from the LC-MS and LC-ESI-MS/MS analysis conducted on crude and enzyme digested duct segments. All except μ-TIIIA and TVIIA were analysed from the venom duct proteome.

Peptide name	Literature mass (Da)	Experimental mass (Da)	MS/MS evidence	[1]Duct segment
ρ-TIA	2389.14	2390.5351<br>2389.2206	-	DC<br>PC
μ-TIIIA	2455.97	-	-	-
TVIA	2844.05	-	Yes	PC
TVIIA	3209.276	-	-	-
Conantokin-T	2682.21	2682.3514	Yes	D
Conopressin-T	1107.53	1107.5675	Yes	P and PC
T1.1	1904.68	1904.2497	-	P and PC
T1.2	1953.78	1953.2469	Yes	D
T6.1	3731.64	-	Yes	PC

[1]*Distal (D), Distal Central (DC), Proximal Central (PC) and Proximal (P).*

S4 Table. List of the major gene superfamilies expressed in the venom duct transcriptome of S1 and S2. The total (T) number of precursors for each superfamily (SF) / specimen (S1/S2), the number of common (C) precursors and representative major precursor information for each superfamily has been detailed out. Additionally, novel precursor information from minor superfamilies has been provided.

SF	T		C	Signal sequence	Mature sequence	Framework	Closest known precursor	[1]Known/Predicted pharmacology	Ref
	S1	S2							
B1	208	191	29	MHLYTYLYLLVPLVTFHLILGTG	GEEEYQKMLENLKRKQES	N/A	Con-T	*NMDA antagonist	[1]
B2	24	11	2	MLRLIIAAVLASACLAY	GEELEERSHHSKFNGDSDNSPFQSEDGLENFMDFMKDNSNE NLPLQQR	N/A	G066_VD	n.d	[2]
O1	90	22	11	MKLTCVVIVTVLLLTAC	CLSPGSSCSPTSYNCCRSCNPYSRKCR	VI/VII	TVIA	*ω /voltage-gated calcium channel	[3]
				MKLTCVVIVAVLLLTAC	QVSWWCGKPEATCGKLYLKCCSGRCNKANWKCL	VI/VII	T6.1	ω /voltage-gated calcium channel	This study
				MKLTCVLIIAVLFLMAC	SRSCSGRDSRCPPVCCMGLMCSRGKCVSIYGEK	VI/VII	TVIIA	Sodium channel	[4]
				MKLTSALIVAVLFLTA	NCFPNGKFCGFPKVGKPCCSGVCLFACT	VI/VII	T6.2	ω /voltage-gated calcium channel	This study
O3	68	17	6	MSGLGIMVLTLLLLVSMA	SRPKTKECERYCELEEKHCCCIRSNGPKCSRICIFKFWC	n.d	T6.3	voltage-gated calcium/sodium channel	This study
				MSGLGIMVLTLLLLVLMTTSH	CEMQCEQKKKHCCRVREERIQCAPKCWGIEW	VI/VII	T6.4	ω /Voltage-gated calcium channel	This study
A	42	19	8	MGMRMMFTVFLFVVLA	FNWRCCLIPACRRNHKKFC	I	TIA	*α_{1B}-noradrenergic	[5]

								receptor antagonist	
				MGMRMMFTVFLLVALA	GCCSHPACSGNNPEFCRQ	I	T1.1	α/nAChR	This study
				MGMRMMFTVFLLVVL	GCCSNPACAGNNPHVCRQ	I	T1.2	α/nAChR	This study
				MGMRMMFTVFLLVVLA	GEPVPTTIINFGECCRDPSCWVKVKDFKC	I	T1.3	α/nAChR	This study
Con-ikot-ikot	29	21	8	MAMNMSMTLSTFVMVVVAAT	TPPPHNDCCKMKMCCAIKTEECLKTHSDQQHVYITICYQEA SHTCGQYNEIVGCCYGYRNCMLINVQQLGLRQAQQTCSNRN CLNPCQ	n.d	Con-ikot-ikot-G2	*AMPA receptor	[6]
Conopressin	24	6	4	MTRSVMQMGRLTLVLCLLLLLLL TTQ	CYIQNCLRV	n.d	Conopressins-T	*Vasopressin-oxytocin receptor	[7]
S	4	2	1	MMSKMGAMFVLLLLFTLASSQ	GCTGNCDWTCSGDCSCQGTSDSCHCIPPKSIGNRCRCQC	VIII	T8.1	Serotonin receptor	This study
M	1	2	1	MMSKLGVLLTICLLLFPLTAL	HGCCKGPKGCSSRECRPQHCC	III	TIIIA	*μ/Sodium channel	[8]

[1]Predicted pharmacology based on the conopeptide class and superfamily.

* Pharmacology has been characterised.

N/A not applicable

n.d not determined.

S5 Table. List of the ρ-TIA precursor variants analysed from the S1 transcriptome. The transcript number, signal sequence, mature peptide sequence and cysteine residue information has been detailed out. All the precursors display a tight conservation of the signal sequence region, whereas sequence variation is observed in the C-terminal sequence of mature peptide, with reference to the ρ-TIA peptide. Most TIA variants have retained the N-terminal sequence (FNWR) which comprises of the TIA pharmacophore interacting with the α_{1B}-adrenoceptor.

Transcript Number	Read frequency	Signal sequence	Mature sequence	Cysteine Number
Tu052/TIA	2	MGMRMMFTVFLFV VLATT	FNWRCCLIPACRRNHKKFC	4
Tu377	3	MGMRMMFTAFLFV VLATT	FNWRCCLIPACRRNHKSFVADDADAHS CHQNNQDMC	5
Tu378	2	MGMRMMFTVFLFV VLATT	KCCSIPKCYKNNKKMC	4
Tu379	2	MGMRMMFTVFLFV VLATT	KCCSIPKCYKKQ	3
Tu380	2	MGMRMMFTVFLFV VLATT	KCCSIPKCYKNNKNKC	4
Tu381	2	MGMRMMFTVFLFV VLATT	FNWRCCLIPAYRRNHKKFC	3
Tu395	3	MGMRMMFTVFLFV VLATT	KCCSIPKCYKNNKKCVADDAGAHSCHQ NNQDMC	6
Tu393	2	MGMRMMFTVFLFV VLATT	FNWRCCLIPACRRNQLKVC	4
Tu396	3	MGMRMMFTVFLFV VLATT	FNWRCCLIPACRRNHKKFVADDADAHS CHQNNQDMC	4
Tu397	4	MGMRMMFTVFLFV VLATT	FNWRCCLIPACRRNHKKFCDSYDADAH SCHQNNQDMC	5
Tu400	4	MGMRMMFTVFLFV VLATT	FNWRCCLIPACRRNHKKFVADDADAHS CHQNKTFCDSYDRQE	4
Tu403	4	MGMRMMFTVFLFV VLATT	FNWRCCLIPACRRNHKKFCGLTTLMLI PVIRTIKTCVA	4

S6 Table. Venom duct transcriptome expression of gene superfamilies in *C. tulipa* and *C. geographus*. Transcriptomic data for both specimens retrieved from 454 pyrosequencing [2]. ✓denotes gene superfamily is present.

Gene superfamily	*C. tulipa* S1	*C. tulipa* S2	*C. geographus*
A	✓	✓	✓
B1(conantokins)	✓	✓	✓
B2	✓	✓	✓
O1	✓	✓	✓
O2		✓	✓
O3	✓	✓	✓
M	✓	✓	✓
S	✓	✓	✓
I1	✓	✓	✓
I3			✓
H	✓	✓	
J			✓
T		✓	✓
Con-ikot ikot	✓	✓	✓
conkunitzin	✓	✓	✓
Conopressin-conophysin	✓	✓	✓
contryphan			✓
contulakin			✓
Newgeo 1	✓	✓	✓
Newgeo2			✓
Newgeo 3	✓	✓	✓
NewGeo 4			✓

References

1. Haack, J.A.; Rivier, J.; Parks, T.N.; Mena E.E.; Cruz, L.J.; Olivera, B.M. Conantokin-T. A gamma-carboxyglutamate containing peptide with N-methyl-d-aspartate antagonist activity. *J Biol Chem* **1990**, *265*, 6025-6029, doi:Retrieved from https://www.ncbi.nlm.nih.gov/pubmed/2180939.
2. Dutertre, S.; Jin, A.H.; Vetter, I.; Hamilton, B.; Sunagar, K.; Lavergne, V.; Dutertre, V.; Fry, B.G.; Antunes, A.; Venter, D.J., et al. Evolution of separate predation- and defence-evoked venoms in carnivorous cone snails. *Nature Comm* **2014**, *5*, 3521, doi:https://doi.org/10.1038/ncomms4521.
3. Chung, D.; Gaur, S.; Bell, J.R.; Ramachandran, J.; Nadasdi, L. Determination of disulfide bridge pattern in ω-conopeptides. *Int J Pept Protein Res* **1995**, *46*, 320-325, doi:https://doi.org/10.1111/j.1399-3011.1995.tb00604.x
4. Hill, J.M.; Atkins, A.R.; Loughnan, M.L.; Jones, A.; Adams, D.A.; Martin, R.C.; Lewis, R.J.; Craik, D.J.; Alewood, P.F. Conotoxin TVIIA, a novel peptide from the venom of *Conus tulipa* 1. Isolation, characterization and chemical synthesis. *Eur J Biochem* **2000**, *267*, 4642-4648, doi:https://doi.org/10.1046/j.1432-1327.2000.01508.x.
5. Sharpe, I.A.; Gehrmann, J.; Loughnan, M.L.; Thomas, L.; Adams, D.A.; Atkins, A.; Palant, E.; Craik, D.J.; Adams, D.J.; Alewood, P.F. Two new classes of conopeptides inhibit the α1-adrenoceptor and noradrenaline transporter. *Nature Neurosci* **2001**, *4*, 902-907, doi:https://doi.org/10.1038/nn0901-902.

6. Hu, H.; Bandyopadhyay, P.K.; Olivera, B.M.; Yandell, M. Elucidation of the molecular envenomation strategy of the cone snail *Conus geographus* through transcriptome sequencing of its venom duct. *BMC Genomics* **2012**, *13*, 284, doi: https://doi.org/10.1186/1471-2164-13-284
7. Dutertre, S.; Croker, D.; Daly, N.L.; Andersson, A.; Muttenthaler, M.; Lumsden, N.G.; Craik, D.J.; Alewood, P.F.; Guillon, G.; Lewis, R.J. Conopressin-T from *Conus tulipa* reveals an antagonist switch in vasopressin-like peptides. *J Biol Chem* **2008**, *283*, 7100-7108, doi:https://doi.org/10.1074/jbc.M706477200.
8. Lewis, R.J.; Schroeder, C.I.; Ekberg, J.; Nielsen, K.J.; Loughnan, M.; Thomas, L.; Adams, D.A.; Drinkwater, R.; Adams, D.J.; Alewood, P.F. Isolation and structure-activity of μ-conotoxin TIIIA, a potent inhibitor of tetrodotoxin-sensitive voltage-gated sodium channels. *Mol Phamacol* **2007**, *71*, 676-685, doi:https://doi.org/10.1124/mol.106.028225.
